# Telemedicine in the Satellite Dialysis Unit: Is It Feasible and Safe?

**DOI:** 10.3389/fmed.2021.634203

**Published:** 2021-04-29

**Authors:** Sabrina Haroon, Titus Lau, Gan Liang Tan, Andrew Davenport

**Affiliations:** ^1^National University Hospital, Singapore, Singapore; ^2^Department of General Medicine, Sengkang General Hospital, Singapore, Singapore; ^3^University College London (UCL) Centre for Nephrology, Royal Free Hospital, University College London, London, United Kingdom

**Keywords:** telemedicine, dialysis unit, safety, quality, hemodialysis

## Abstract

Telemedicine has gained popularity during the recent COVID-19 pandemic. Regular and timely physician review is an essential component of care for the maintenance of hemodialysis patients. While it is widely acknowledged that telemedicine cannot fully replace the role of physical review in this group of patients with organ failure, it can perhaps reduce the reliance on physical review or serve as a filter and triage in determining which patient requires actual physical review. The use of technology in any healthcare setting should always align with existing clinical workflow and protocols. We discuss the safety and quality aspects of this new concept applied to the satellite dialysis unit.

## Introduction

Telemedicine is defined as the use of electronic information and communication technologies to provide and support healthcare-related activities remotely when distance separates the participants (1). In response to the recent COVID-19 pandemic, some dialysis providers have transitioned from physical review to review via telemedicine to minimize patients' and staffs' movement as a strategy to reduce the risk of transmission. While the demand and introduction of telemedicine would appear appropriate and acceptable for many clinical services, including home hemodialysis (HHD) and peritoneal dialysis (PD), where patients perform self-care at home, this is a relatively new concept in the management of satellite hemodialysis units (2–4).

## Operations of a Satellite Dialysis Unit and Risk of COVID-19

In a satellite hemodialysis unit, the number of dialysis stations can range from as few as five up to sixty stations or more, operating up to three shifts a day with healthcare staff entirely or partially performing patients' dialysis treatments. On average, patients usually spend a minimum of 5 h in the dialysis unit from arrival, screening, preparation, actual dialysis, and ending of treatment. A dialysis unit's operations involve the physician who may not necessarily be a nephrologist depending on the country's regulatory requirement, nurses, dialysis technicians, administrative staff, and housekeeper/domestic staff. Depending on the dialysis landscape, some satellite units are located within hospital premises, whereas others are in remote areas and may require traveling times of more than 1 h. The staff-patient ratio varies from one to three in higher acuity units down to one to seven in more stable units. This is often dependent on the number and level (either nursing or dialysis technician) of healthcare staff onsite at the unit (5, 6). Dialysis practices differ across continents and countries, with physical review by nephrologists varying from every dialysis session, to weekly visits, monthly or even longer interval reviews.

Corbett et al. reported clustering of cases of Covid-19 in specific dialysis units and specific shifts associated with high nursing staff illness rates. The risk of COVID-19 transmission in the dialysis unit is high given the older age and multiple co-morbid conditions in end-stage renal disease (ESRD) patients, regular contact with healthcare staff, shared common transportation, duration of dialysis treatment, and proximity to the next patient with the usual distance of <2 meters in general. The transmission in the dialysis unit can occur between patient to patient, staff to patient, physician to the patient, and vice versa. Telemedicine will not necessarily reduce patient-to-patient transmission, but the transmission from staff-to-patient is lowered with protection bias favoring physicians compared to other healthcare workers (7, 8).

A single nurse usually works for 2–3 dialysis shifts, handling up to a maximum of 7 patients per shift in the day. In this situation and with personal protection equipment (PPE) compliance, the transmission risk is limited mainly to one dialysis nurse. In contrast to the physician, where the contact between the physician and the patient is generally less frequent, shorter, and of lower “intensity,” occurring mostly during regular rounds. Thus, the risk will be assumed to be lower. Although, one may argue that telemedicine may only offer bias protection mainly for the physician, the movement of the physician and the possible acquisition from asymptomatic patients at the dialysis unit can still occur. The physician often has other additional duties which may result in “spreading” to other areas of clinical work.

## Substituting Telemedicine Review for Physical Review

ESRD patients generally have a high co-morbid burden and require dialysis to sustain life. Regular and timely physician review is an essential component of the standard of care for maintenance hemodialysis patients. An international comparative study reported a positive association between the frequency of physician visits and contact time with ESRD reduced mortality and hospitalization (9). The conventional in-person review of dialysis patients generally involves the nephrologist visiting the dialysis unit at certain prescribed intervals and conducting patient rounds at the unit. Telemedicine review cannot fully replace the role of physical review in this group of patients with organ failure with multiple co-morbidities, but perhaps it can reduce the reliance on physical review or serve as a filter and triage in determining which patients require actual physical review. For example, telemedicine or virtual real-time review via live audio-visual interactive session cannot replicate the physical examination needed to accurately diagnose some of the more common problems associated with maintenance hemodialysis patients, such as fever, abdominal pain and chest pain to accurately make diagnosis and treat. Thus, there will be a need for an actual physical review at specific time points or when new symptoms arise that demand a physical examination be conducted for diagnostic purposes. To further reduce the need for wasteful and unneeded consults, additional measures should be put in place to coordinate care to reduce repetition if the physician reviewing the patient in the dialysis unit is different from the primary nephrologist at the hospital. In such cases, when a physical review is necessary, the review can be done either at the dialysis unit or at the hospital, whichever reduces the risk of potential exposure to both patient and clinician.

As a response to the current COVID-19 pandemic situation, telemedicine review can take the form of a real-time two-way audio-visual interactive session to replace the conventional physical review visits at satellite dialysis units Other routinely captured treatment and patient parameters can also be made available electronically and transmitted in real-time (remote monitoring) or via store-and-forward mode at the appropriate time and interval. The concept of remote monitoring started in satellite dialysis units in the late 1980s, before the introduction of high-speed internet. Bernstein et al. reported their experience of remotely managing hemodialysis units and compared the clinical outcomes with local routine in-person hemodialysis care (10). The authors found equivalent survival rates and possibly even better outcomes in the remotely managed group compared with the routine care cohort followed up-to 5-years (10). While it is widely acknowledged that telemedicine cannot fully replace in-person physical review, it can perhaps reduce the frequency of such reviews. However, the scarcity of published evidence on its effectiveness and safety for utilization in this group of patients is of significant concern. We suggest programs adopting telemedicine in satellite dialysis units to evaluate and ensure the quality and safety of such a practice, even if it is an intuitive approach during an infectious crisis.

Potential areas for telemedicine would mainly be in the management of stable asymptomatic established dialysis patients. The review of information about inter-dialytic weight gains and trends in serial weights, peri-dialytic blood pressures, may aid the clinician in determining volume status without recourse to physical review. The role of natriuretic peptides and bioimpedance estimates of extracellular water can be considered in borderline cases depending on availability, as an adjunct to standard investigations (11). In addition, pictures or short videos of an arteriovenous fistula, with the results of a bedside doppler examination of the fistula performed by a nurse or technician, swollen lower limbs or views of the jugular venous pulse or neck swelling can also be viewed via a telemedicine platform.

The decision and feasibility of telemedicine review compared to physical review depend on several key factors (Table 1). One of telemedicine's fundamental requirements is the availability of the necessary technological infrastructure to ensure effective patient and clinician communication. We will discuss in the subsequent sections specific considerations when implementing telemedicine review in the satellite dialysis unit. The type and frequency of these reviews will depend on several factors. For example, in those dialysis units that cater to more frail and highly co-morbid patients, there should be greater emphasis on the physical review given the complexity and reduced cardiovascular stability of these patients.

**Table 1:** Factor determining the frequency of physical review.

Geographic access (12)
Patient characteristics and profile
Availability of on-site nurse clinician or advance practice nurse
Availability of adjunct dialysis tools
Availability of IT infrastructure for adoption of telemedicine

Patients at the satellite dialysis unit have a minimum of twice-weekly contact with trained healthcare staff, who will be able to perform basic clinical assessments and counseling, and then report to the physician in charge. The availability of trained nurse clinicians or advanced practice nurses will help supplement physician review as they are better trained and equipped to undertake higher-level clinical management responsibility in addition to the essential management of dialysis patients. Advances in technology and clinical research have introduced tools including bioimpedance, transonic ultrasound, and blood biomarkers such as brain natriuretic peptide and beta2 microglobulin to guide and complement clinical assessment. However, these tools are not universally available in all dialysis units.

## The Process of a Telemedicine Review

We suggest some of the following measures in adopting telemedicine in satellite dialysis units.

### Conduct of Telemedicine Review

Basic regular telemedicine procedures and processes are necessary. As there are various parameters, dialysis flowsheets, laboratory investigations, and other non-clinical issues to address for ESRD patients treated with hemodialysis, there should be a standardized workflow or process to help direct how the session should be conducted. The conduct of a telemedicine review can mirror that of the usual in-person review. There are, however, additional considerations to consider while adopting telemedicine to dialysis units since patients are receiving dialysis treatment during their time at the unit.

To avoid overcrowding and maintain compliance with social distancing measures, the review should be completed as efficiently as possible, avoiding unnecessary queuing and waiting time. We suggest a detailed pre-review of cases and meeting with the nursing team before the actual scheduled telemedicine session with patients to increase efficiency and allow the dialysis session to be completed in a timely manner.

Dialysis sessions can be eventful, with occasional fluctuations in blood pressure and accompanying symptoms. Multiple alarms during treatment can disrupt the sessions, and there will be a need to safeguard privacy given the proximity to other patients in neighboring stations. Conducting a review post-dialysis may be compromised by post-dialysis fatigue, which is not uncommon. Therefore, the ideal time to conduct a telemedicine review is before starting treatment in a private consultation room. Many elderly end-stage renal failure patients may have hearing impairment, poor vision, and a high co-morbid burden; hence, the availability of information technology (IT) assistive devices such as headsets and microphones should be made available. Furthermore, these co-morbidities (such as blindness or hearing impairment) may also limit the use of telemedicine or even make it impossible. There may also be a need to address linguistic minorities with limited language proficiencies by having specific interpreters available for the session, although internet oral translational services are expanding the range of languages and becoming more readily available (13). However, it needs to be recognized that additional time will be needed for telemedicine consultations for patients with communication difficulties. While telemedicine reviews aim to reduce the reliance on physical review, all units should have an established escalation workflow that dictates when a patient receiving telemedicine reviews should be transitioned to urgent-in-person follow-up care or even to emergency services directly (13).

### Patient Information and Data Security

There are various IT platforms and software vendors or providers in telemedicine, and we emphasize here the role of cybersecurity and the need for monitoring data accessibility and security given the large volume of data available online. Software and hardware must be practical, sustainable, and all necessary data should be visible to the physician. Newer generation dialysis machines can be linked to a network to provide online treatment data, which can be reviewed live during the consultation or retrieved as required. Any other data not available online, such as home monitoring documents, should also be uploaded before the session (13).

All telemedicine platforms must adhere to the local healthcare service act or regulations to ensure compliant technical specifications (14). A few standalone software programs may be used in telemedicine, including Zoom for Healthcare, Skype for business, and the Smart Health Video Consultation platform (15). Before adopting telemedicine, providers will be required to go through a telemedicine e-training module covering the use, limitations, and implementation of telemedicine, including video consultation. Patient consent will need to be obtained and counseled on telemedicine's role, emphasizing that it is not meant for emergency consultation (16).

During this session, the focus is on video consultation, one of the synchronous telemedical consultations. A structured video consultation should involve considering the environment, session initiation, dialogue, and session closure (17). A separate room that ensures privacy will be ideal, with uninterrupted internet access to conduct the video consultation. Once the call is established, a quick check should be made to ensure that sound and picture quality are satisfactory (17). The location of the provider and patient should be checked and written down during the session. Once the session ended, the consult notes should be completed for future correspondence.

Other areas to consider are electronic health record security, data protection, and privacy issues. To avoid subjecting patients to companies' privacy policies, it is reasonable to consider a service agreement such as Business Associate Agreements (BAA). However, this requirement will significantly raise the costs and complexities of telehealth software investments. An approach to encourage companies to sign binding service agreement is to make this known publicly if they comply with specific standards such as Health Insurance Portability and Accountability Act (HIPAA). Such an approach would incentivize platforms to comply with HIPAA, as it would be in companies' business interest to gain public credibility and trust that would encourage more consumers to adopt their technology and use their services (18).

### Other Responsibility of the Medical Director

In addition to clinical management, the nephrologist in charge may also be the unit's designated medical director. The medical director has mandated responsibilities in governance and oversight of the unit, including monitoring safety issues, risk of falls, fire safety, conducting audits, participating, or leading root cause analysis/incident analysis (19, 20). It is certainly not possible to fulfill this role with interface technology alone. Some options may be possible and appropriate, such as reviews of standardized reports, auditing via closed-circuit television (CCTV) footage, and small group discussion can take the form of a live interactive audio-visual session to reduce the need to meet in person and movement of staff. However, some activities, such as taking samples to check on water quality, cannot be fully replaced by the current IT platform.

## Measuring Safety and Quality of Telemedicine in the Dialysis Unit

The introduction of telemedicine into the dialysis unit is a relatively new concept with limited evidence in patient safety and quality measurement, both in the short and long term. We suggest the following measures as dialysis programs adopt increasing use of telemedicine in the dialysis unit.

Monitoring hospital admissions and comparison with previous historical data is vital. Specifically, admissions due to volume or vascular access problems are clinically relevant as these are key areas for examination and review during rounds at the dialysis unit. The presence of adjunct tools to assess patient volume status and vascular access should theoretically help improve the review. However, the recently published Kidney Disease Quality Initiatives (KDOQI 2019) on Vascular Access emphasized the need for regular physical examination of the arteriovenous (AV) access, although this is within the remit of experienced dialysis nurses (21). Thus, telemedicine review supplemented by an experienced nurse should not compare unfavorably to physical rounds.

The introduction of any new technology may raise concerns with staff as to whether telemedicine will increase workloads and impact their ability to perform their traditional tasks and responsibilities (22). Some dialysis unit staff may not be comfortable managing without the physician's physical presence for extended periods or when clinical decisions are made online, so the path of communication and documentation should be precise and clear. Clinicians equally may have apprehensions for the time allocated for each patient and a sense of incompleteness without physically examining the patient. We suggest using “Telehealth Consultant Satisfaction Survey” to examine providers' perception of telemedicine (23).

The use of telemedicine has been shown to improve the KDQOL-SF score among PD patients (24). It also helped reduce the depression, anxiety, and stress score (DASS) among hemodialysis patients (25). However, there are still limited data on patients' views on replacing physical review with video consultation, although the “Telehealth Patient Satisfaction Survey” has been widely used to assess the satisfaction of replacing physical review with telemedicine (26). It may take a little more time and assurance for all stakeholders, especially older patients, less exposed to the virtual world, to accept this as a standard channel of a health review.

Telemedicine use has been reported to save cost and time needed to travel for the care provider and patient to meet in person, especially for those who live in rural communities (27, 28). However, cost and time saving using telemedicine for patients requiring in-center dialysis treatment two to three times a week in a center sited in an urban settlement are still unclear. Programs adopting telemedicine use in the dialysis unit should evaluate cost-effectiveness along with quality indices (Table 2).

**Table 2:** Measuring safety and quality indices of telemedicine in dialysis unit.

Level of participation of various stakeholders
Hospital admission for volume excess and vascular access issues
Health outcomes
Staff satisfaction survey
Patient satisfaction/patient reported outcome measures
Cost effectiveness
Time spent on telemedicine

## Conclusion

For many dialysis physicians and medical directors, the decision has always been easiest to default to the standard practice of physical rounds. However, with the recent COVID pandemic, telemedicine has gained increasing popularity and significance. We feel that telemedicine is complementary and can reduce the frequency of physical rounds. The use of technology in any healthcare setting should always align with existing clinical workflow and protocols, taking into consideration the physician's level of comfort in managing various clinical conditions and non-clinical situations. While it has proven to be safe, accurate, and reliable in some clinical settings, both short term and long-term outcome validations for in-center dialysis patients are necessary. Information technology will shape and continue to evolve many aspects of medical practice. However, the central focus of a physician's presence is vital in the process of healing, and hence, humanity must remain embedded in this cycle of change and adaptation.

## Author Contributions

The manuscript was drafted by SH and GT. The manuscript was revised by TL and AD. All authors approved the final version of the manuscript and are accountable for the author's own contributions, accuracy or integrity of the manuscript.

## Conflict of Interest

The authors declare that the research was conducted in the absence of any commercial or financial relationships that could be construed as a potential conflict of interest.

## Abbreviations

COVID-19: Coronavirus 2019
HHD: home haemodialysis
PD: peritoneal dialysis
ESRD: End-stage renal disease
PPE: Personal protection equipment
IT: information technology
BAA: Business Associate Agreements
HIPAA: Health Insurance Portability and Accountability Act
CCTV: closed-circuit television
AV: arteriovenous
KDOQI: Kidney Disease Quality Initiatives
KDQOL-SF: Kidney Disease Quality of Life Short Form
DASS: depression, anxiety and stress score.
